# Digital light processing printed hydrogel scaffolds with adjustable modulus

**DOI:** 10.1038/s41598-024-66507-x

**Published:** 2024-07-08

**Authors:** Feng Xu, Hang Jin, Huiquan Wu, Acan Jiang, Bin Qiu, Lingling Liu, Qiang Gao, Bin Lin, Weiwei Kong, Songyue Chen, Daoheng Sun

**Affiliations:** 1 Pen-Tung Sah Institute of Micro-Nano Science and Technology, Xiamen University, Xiamen, 361102 China; 2 Guangdong Provincial People’s Hospital, Guangzhou, 510080 China; 3 Guangdong Beating Origin Regenerative Medicine Co. Ltd, Foshan, 528231 Guangdong China

**Keywords:** Digital light processing, Double network hydrogels, Hydrogel scaffolds, Adjustable modulus, Biomedical engineering, Soft materials, Tissue engineering

## Abstract

Hydrogels are extensively explored as biomaterials for tissue scaffolds, and their controlled fabrication has been the subject of wide investigation. However, the tedious mechanical property adjusting process through formula control hindered their application for diverse tissue scaffolds. To overcome this limitation, we proposed a two-step process to realize simple adjustment of mechanical modulus over a broad range, by combining digital light processing (DLP) and post-processing steps. UV-curable hydrogels (polyacrylamide-alginate) are 3D printed via DLP, with the ability to create complex 3D patterns. Subsequent post-processing with Fe^3+^ ions bath induces secondary crosslinking of hydrogel scaffolds, tuning the modulus as required through soaking in solutions with different Fe^3+^ concentrations. This innovative two-step process offers high-precision (10 μm) and broad modulus adjusting capability (15.8–345 kPa), covering a broad range of tissues in the human body. As a practical demonstration, hydrogel scaffolds with tissue-mimicking patterns were printed for cultivating cardiac tissue and vascular scaffolds, which can effectively support tissue growth and induce tissue morphologies.

## Introduction

Tissue scaffold plays an important role in tissue engineering, providing structural support for the biomimetic characteristics of tissues^1^. The main motivation is to replicate the specific structures and mechanical environments of human tissues, thereby promoting effective tissue remodeling^2^. However, the morphology and modulus of soft tissues vary widely within the human body, e.g. liver (modulus < 10 kPa^3,4^) possesses functional hepatic lobules arranged in a radial distribution, cardiac tissue (modulus ~ 10 kPa^5–10^) exhibits an organized orientation, blood vessels (modulus ~ 10^3^ kPa^11^) feature network-like structures. Moreover, the modulus of cardiac tissue increases with development, starting at approximately 12 kPa in the embryonic stage and reaching around 30 kPa in adulthood^12^. This makes the manufacturing of diverse tissue scaffolds challenging while aiming to match the complexed structural and mechanical properties.

Hydrogels find extensive applications in tissue engineering, due to their excellent properties including flexibility, stretchability, and biocompatibility^13–16^. UV-curable hydrogels, in particular, offer a versatile solution for creating high-resolution 3D structures through techniques like Stereolithography or Digital Light Processing (DLP)^17–21^. It is feasible to efficiently and rapidly manufacture bionic tissue scaffolds with macro-microscopic structures. For example, tissue scaffolds with structures like networked blood vessel scaffolds^22,23^, tendon-like scaffolds^24^, and irregular-shaped bone and cartilage engineering scaffolds^25,26^ can be manufactured using UV-curable hydrogel in DLP fabrication^27^. And DLP-printed hydrogel scaffolds can easily replicate the specific structures of diverse tissue.

Currently, the mechanical properties of hydrogel scaffolds can be generally modulated through UV exposure control^28,29^, structural design^30^, and formula adjustment^23,31^. UV energy intensity and exposure time have a pronounced effect on the mechanical behavior of hydrogels^28,29^. However, unavoidable scattering, transmission, and refraction of light occur during printing, resulting in varying crosslinking densities across different parts of the scaffold (where monomers are not fully crosslinked). This becomes more pronounced in scaffolds with complex structures^27,29^. Structural design is another important way to modify the modulus of a scaffold. Incorporating hollow structures into the scaffold reduces the overall stiffness of a sample^28,30^. The cells sense the local modulus of scaffolds, which indicates a structural resolution of micrometer scale is required. Such methods would greatly reduce the efficiency and raise the cost of printing^32,33^.

By adjusting the components or ratios in the formula, the polymer network and the modulus of the DLP-printed sample can be regulated remarkably^23,31^. An increase in the monomer content or the number of unsaturated bonds in the formula can raise the crosslinking density of the polymer network rapidly^29,34^. On the contrary, if components prone to hydrolysis are added to the polymer network, the crosslinking density of the polymer network decrease, which results in a reduction of the modulus^35^. The presence of additives in solid form into the polymer network will also enhance the sample modulus. The addictive and hydrogels form composite materials, and the cured sample exhibited higher modulus with increasing the additive contents^36^. However, scaffolds produced with the same formula have a limited modulus adjusting range, making it challenging to match the mechanical environment required by a variety of tissues. On the other hand, altering the formula reduces versability, raises printing costs, and lowers its maneuverability.

Herein, we report a two-step process to realize simple adjustment of mechanical modulus over a broad range, by combining DLP and post-processing steps. In contrast to methods that require adjustment of formulas or processes, this two-step process allows broad modulation of modulus using a single UV-curable hydrogel formula and DLP process parameters. Through this two-step process, hydrogel scaffolds with exceptional resolution, biomimetic microstructural, and adjustable modulus were fabricated. These attributes represent a distinctive advantage not attainable through other bioprinting processes. The fabricated scaffold, mirroring the morphology and modulus of native tissue, effectively induced the cultivation of well-organized, synchronized beating cardiac tissue or branching coronary artery. Importantly, this manufacturing process is user-friendly and broadly applicable to ion-crosslinked double network hydrogel systems, and is expected to provide an important method for biomimetic tissue engineering.

## Experimental

### Materials

Acrylamide (AAm) (Macklin, Shanghai, China) as the monomer; poly(ethylene glycol) diacrylate (PEGDA, Mw = 1000) (Macklin, Shanghai, China) as crosslinker. Sodium alginate (Alg) (Rhawn, Shanghai, China) was introduced as a second crosslinking network, Lithiumpheny-2,4,6-trimethyl-benzoylphosphinate (LAP) (Energy Chemical, Shanghai, China) photoinitiator, Tartrazine (Aladdin, Shanghai, China) as a UV absorber. FeCl_3_·6H_2_O (Acmec, Shanghai, China) was used as ionic crosslinks to adjust the modulus of the sample. Deionized water (DI water) was produced for laboratory pure water systems (Master-Q30UT, HHitech, Shanghai, China).

### Preparation of UV-curable hydrogel solutions

The proposed UV-curable hydrogel formula was a mixture of AAm, PEGDA, and Alg precursors. The basic printing solution was developed with a composition of AAm: PEGDA: LAP: Tartrazine: DI water = 1: 0.03: 0.03: 0.015: 4. The hydrogel solution was prepared at five concentrations of Alg (0, 1, 2, 4, and 6% Alg/AAm ratios). Alg solution was prepared by dissolving quantitative powder in 100 g of DI water under magnetic stirring for 12 h at 35 °C. 0.75 g of LAP and 0.375 g of Tartrazine were dissolved in the Alg solution by stirring for 2 h at 25 °C. 25 g AAm and 0.75 g PEGDA were added to the above mixture solution and stirred for 5 h to obtain the desired UV-curable hydrogel solution. It is worth noting that stirring should be conducted in the dark, and the resulting hydrogel solution should be stored in a refrigerator.

### Hydrogel samples manufacturing

**Figure 1:**
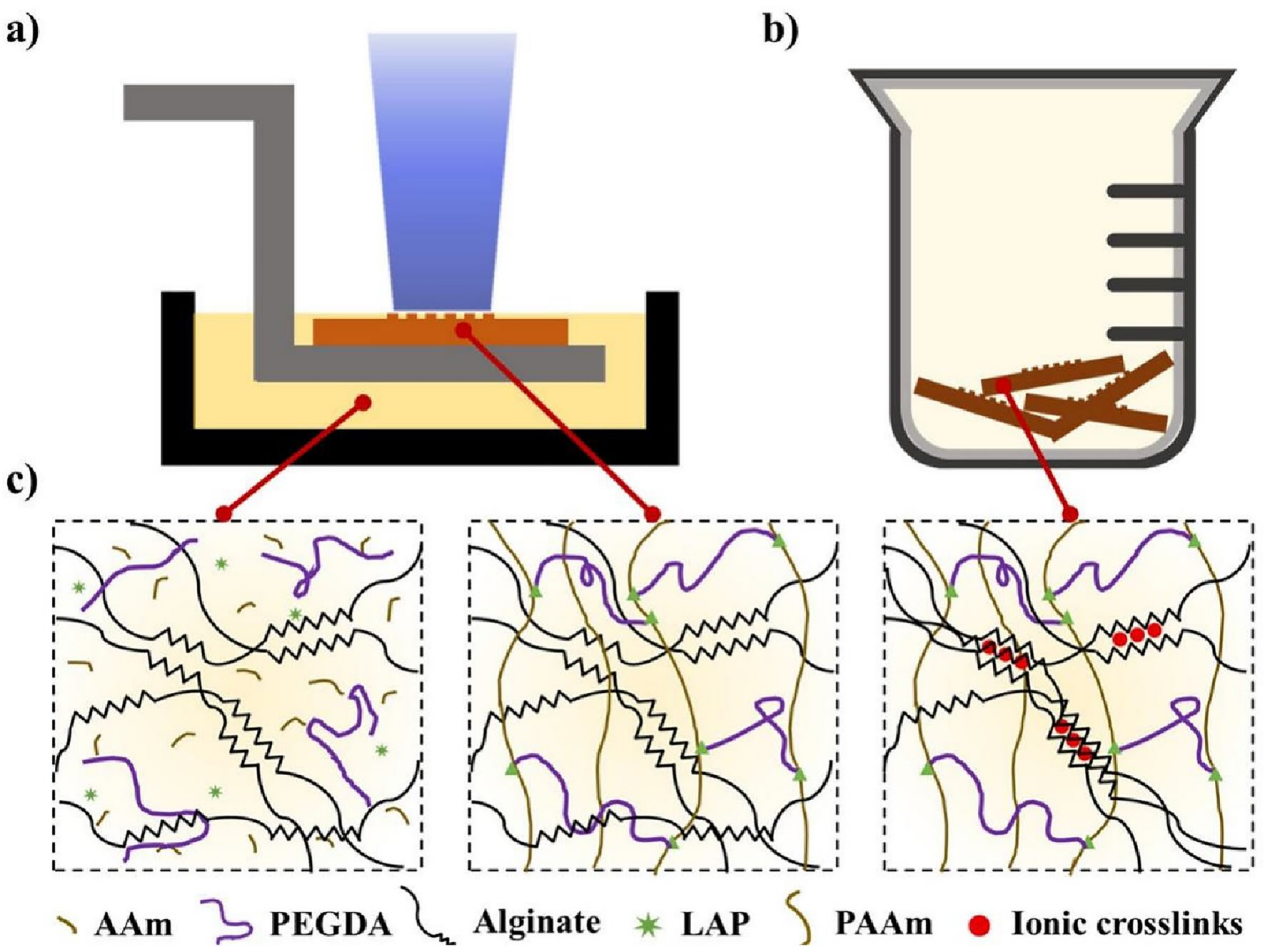
Two-step process: DLP printing and modulus adjustment. (**a**) Schematic diagram of top-down DLP printing. (**b**) Treatment of the sample in an ion bath. (**c**) Crosslinking process of the hydrogel solution: First step, the solution was cured under 405 nm light to form a long-chain polymer network during the DLP process, and the sample was 3D molded at this stage. Second step, the secondary crosslinking of alginate in the samples was performed using Fe^3+^ ion bath treatment. The sample modulus can be regulated by controlling the Fe^3+^ concentration.

The samples were fabricated using a 3D printer (S240, BMF Precision Tech Inc., Chong Qin, China) based on digital light processing technology, which provides a resolution of 10 μm (Fig. 1a). A 405 nm light source was used, and the light energy density was adjusted to 43.1 mW/cm^2^ for our experiments. The 3D digital model was sliced using CHITUBOX V1.9.4, with 10–40 μm for each layer, corresponding to an exposure time of 4–6 s.

The printed samples were immersed in a 40 wt% ethanol solution for 15 min to dissolve any hydrogel solution on the surface. All sample surface was dried using a high-pressure air gun. Then the samples were post-cured under UV light (1000 mW, 15 min). Subsequently, the samples were soaked in a corresponding concentration of Fe^3+^ solution for 24 h to guarantee a complete ion exchange and crosslinking with alginate in the ion bath (Fig. 1b)^37^. To ensure uniform crosslinking, a sufficient amount of Fe^3+^ ion bath solution is provided so as to maintain the stability of the ion concentration during the soaking (Fig. 1c).

### Characterization of hydrogels

The rheological properties of hydrogel solutions were measured using a rotational viscometer (MCR302, Anton Paar, Graz, Austria). All measurements were performed at 25°C (except for temperature scans) and low viscosity solutions were tested using a cone-and-plate geometry of CP50-1 (cone angle: 1°, diameter: 50 mm).

The swelling behavior of hydrogel samples was evaluated using rectangular specimens measuring 16 mm × 10 mm × 2 mm. The lengthwise deformation of the samples was recorded for 7 days after crosslinking with Fe^3+^ at concentrations of 0, 0.005 M, 0.01 M, 0.02 M, and 1 M, for alginate content of 0 wt%, 1 wt%, 2 wt%, 4 wt%, and 6 wt% (Alg/AAm).

At room temperature, the mechanical properties of hydrogel samples were measured using an electromechanical universal testing machine (E43, Meters Industrial Systems, USA) @2 mm/min with a 50 N load cell. Each sample was tested and recorded with a dial caliper with a precision of 0.01 mm. Unless otherwise specified, the hydrogel samples were subjected to ion bath treatment for 1 day and soaked in DI water for 7 days. According to the diffusion formula of ions in an aqueous solution, prolonged soaking allows for the uniform diffusion of Fe^3+^ into the hydrogel samples^37^. The structures of testing models and hydrogel scaffolds were characterized and measured using a digital microscope (DSX1000, Olympus Corporation, Japan).

After ion bath treatment, PAAm-Alg hydrogels were cut into equal-sized pieces (~ 6 mm × 6 mm × 3 mm) for biocompatibility testing. After rinsing thoroughly with phosphate-buffered saline (PBS), the hydrogel samples were incubated in quantitative DMEM medium (the ratio of surface area to medium volume is 3 cm^2^/mL) for 72 h @37 °C. The biocompatibility was tested on fibroblast cells cultured in the extract of hydrogels using Enzyme Markers (K3 Touch, Thermo Fisher, USA), and a CCK-8 kit (Shanghai Beyoncé Bio, C0043) after 72 h.

### Generation of HiPSC derived cardiomyocytes and endothelial cells

HiPSC (Beijing Cel-lapy Biological Technology Co. Ltd., Beijing, China) were cultured on matrigel-coated (0.15%, Corning, 356234) six-well plates in StemFlexTM medium (Gibco, A3349401) at 37 °C in 5% CO_2_. The hiPSCs were labeled with green fluorescent by introducing an EGFP expressional element in an AAVS1 locus. HiPSC-CMs were cultured according to previous publications^38,39^.

Human Coronary Artery Endothelial Cells (HCAEC) (FengHuiShengWu, CL0117) were cultivated as adherent cells in Endothelial Cell Medium (ECM, Sciencell, 1001). When the cell confluence reached 80%, a 0.05% trypsin solution was employed for a digestion period of 8–10 min. To halt the enzymatic digestion, a serum-containing culture medium was used. Subsequently, the cells were gently detached using a pipette, transferred to a centrifuge tube, and subjected to centrifugation at 200*g* for 5 min. After discarding the supernatant, the cell pellet was resuspended in ECM to achieve a concentration of 5 × 10^6^/mL and then seeded onto the hydrogel substrates.

### Seeding and culture of the cardiomyocytes and endothelial cells

All samples underwent sterilization through γ-ray irradiation. The hydrogel scaffolds were immersed in the culture medium three consecutive times, with each immersion lasting no less than 3 h. Subsequently, the samples were immersed in the culture medium for more than 72 h and stored in a refrigerator. Before cell seeding, all hydrogel scaffolds were coated with porcine gelatin (Sigma, V900863) at 2% w/v in phosphate-buffered saline (PBS) for 2 days @37 °C.

Cardiac tissue scaffolds: a cell suspension was prepared by mixing cardiomyocytes and fibroblasts with a ratio of 2:1. 500 μL cell suspension (3 × 10^5^/well) was seeded on a hydrogel scaffold, and all samples were incubated for 12 h @37 °C to allow cell attachment. Subsequently, the devices were cultured in the RPMI 1640 Medium with 3% KnockOut Serum Replacement @37 °C, and 5% CO_2_ every 2–3 days changes of media for the duration of the experiment.

Vasculature-like hydrogel scaffolds: 15 μL of endothelial cell suspension (7.5 × 10^4^/well) were pipetted onto the central groove of the samples. Culture medium was added 4 h later. For immunofluorescence staining, cells were fixed with 4% paraformaldehyde for 20 min, followed by permeabilization with 0.25% Triton-X 100 (Sigma-Aldrich) in PBS for 20 min at room temperature.

The morphology of tissues cultured on the scaffold was recorded using a fluorescence microscope (MF52-N, Mshot, China). Furthermore, rhythmic contractions of tissue were induced using a custom-made electrical stimulation device, employing a pair of electrodes to generate a uniform electric field. Unless otherwise specified, the electrical field strength applied to the cardiac tissue by the stimulation device was set at 8 V/cm. The cardiac tissue contractions were recorded through video documentation.

### Statistical analysis

The data were indicated by the mean ± standard deviation (SD). We conducted swelling, surface morphology, and mechanical testing calculations for each experimental group at least three times. We used OriginPro2017 software for statistical analysis was applied to compare the mean values within each group. The data of Stress–strain curves in the paper has undergone smoothing processing.

### Ethical approval

The cardiomyocytes and endothelial cells used in the study were commercially cells. All cells experiment in the manuscript were approved by the Declaration of Helsinki guidelines.

## Results and discussions

### Printability and swelling behaviors of the hydrogel formula

To meet the desired printability and swelling behaviors, the AAm-Alg hydrogel formula was carefully designed, as shown in Fig. 1. AAm has been extensively studied as a UV-curable hydrogel solution. To minimize the toxicity of printed samples, the hydrogel formula was improved by using LAP and Tartrazine, which have superior biocompatibility. And PEGDA (Mw = 1000) was added as a crosslinker to form rigid chains connecting the flexible chains of polyacrylamide (PAAm), enhancing the mechanical properties of the polymer network^40,41^. Among the variety of choices, a formable formula (AAm: PEGDA: LAP: Tartrazine: DI water = 1: 0.03: 0.03: 0.015: 4) was selected.

**Figure 2:**
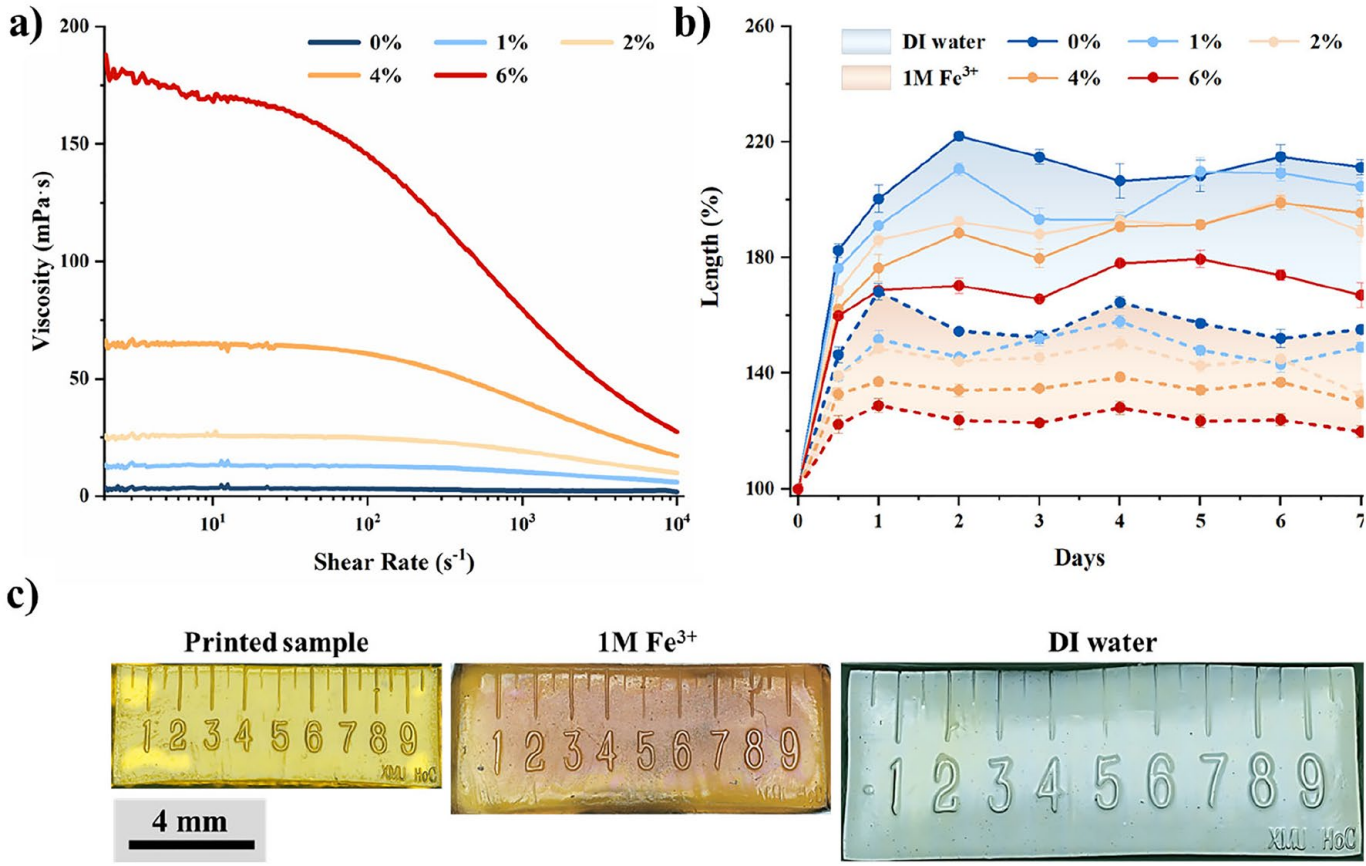
Viscosity and swelling properties of hydrogel. (**a**) Viscosity curves of hydrogel solutions with Alg/AAm ratios of 0–6%. (**b**) Swelling properties of cured hydrogel samples with different Alg/AAm ratios subjected to 1 M Fe^3+^ crosslinking (dashed) or without crosslinking (solid), compared by the lengthwise deformation of the testing samples. (**c**) The swelling of a 4% Alg/AAm ‘Hydrogel Ruler’ in different conditions.

A sufficient fluidity of the UV-curable formula is crucial to reduce the difficulty of the recoating process in DLP printing, thereby improving both the printing efficiency and quality. A hydrogel solution with low viscosity enhances printability, including continuity and thickness control^42,43^. Samples printed with high viscosity hydrogel solution are prone to defects in structures^44,45^. However, to create a dual-network hydrogel with an adjustable modulus, Alg was added into the formula. The addition of alginate increased the viscosity and brought distinct non-Newtonian fluid behavior to the hydrogel solution, as shown in Fig. 2a. At a low shear rate of 10 s^−1^, the viscosity of the hydrogel solution (0–6 wt% Alg/AAm) increased from 3.58 mPa s to 168 mPa s (Fig. 2a). Besides, the rheological properties of this formula are temperature-sensitive. In a temperature scan ranging from 25 to 60 °C, the 4 wt% solution viscosity decreased from 61.4 mPa s to 26.4 mPa s @50 s^−1^ (Supplementary Fig. S1).

In addition, the swelling behaviors of hydrogels are closely related to Alg. Cured PAAm-Alg hydrogels exhibit hydrophilicity due to the abundant hydrophilic functional groups in the polymer chains. Therefore, those cured samples swelled at immersion in solutions, resulting in undesirable shape changes^46^. Excessive deformation is not conducive to printing scaffolds with high resolution. Advanced dual-network hydrogel formula design helps the swelling control. The deformation extent of 0–6 wt% (Alg/AAm) UV-curable hydrogel samples in DI water, 0.005 M, 0.01 M, 0.02 M, and 1 M Fe^3+^ environments was recorded for 7 days. At the same Alg content, the samples treated in different ion baths exhibit similar deformations (Supplementary Fig. S2). However, the swelling deformations ratio of the samples decreased with a higher content of alginate in the same ion bath (Fig. 2b). Notably, the addition of alginate created a double network hydrogel that limited swelling even in DI water without ionic crosslinking. After 1 M Fe^3+^ bath treatment, the swelling deformation of 4 wt% and 6 wt% samples (~ 130%, and ~ 120%, respectively) were comparable and acceptable. Ultimately, balancing the viscosity and swelling requirements of the formula, a UV-curable hydrogel solution containing 4 wt% alginate was selected in this study. To visualize the swelling deformation of hydrogel samples, a 10 mm hydrogel ruler (4 wt% Alg/AAm) was printed. In Fig. 2c, the state of the hydrogel ruler was shown directly after printing, either with 1 M Fe^3+^ bath treatment, or after immersion in DI water. The ratio of their length to width was nearly equal (2.67, 2.53, 2.67 respectively). It could be confirmed that, after swelling, the manufactured hydrogel samples undergo consistent deformation on a macroscopic scale.

It is worth mentioning that the cost-effective UV-curable hydrogel (priced at approximately $10 for 100 g solution) exclusively incorporates readily accessible commercial-grade raw materials, thereby diminishing the entry threshold for 3D bioprinting.

### Optimization of DLP parameters

**Figure 3:**
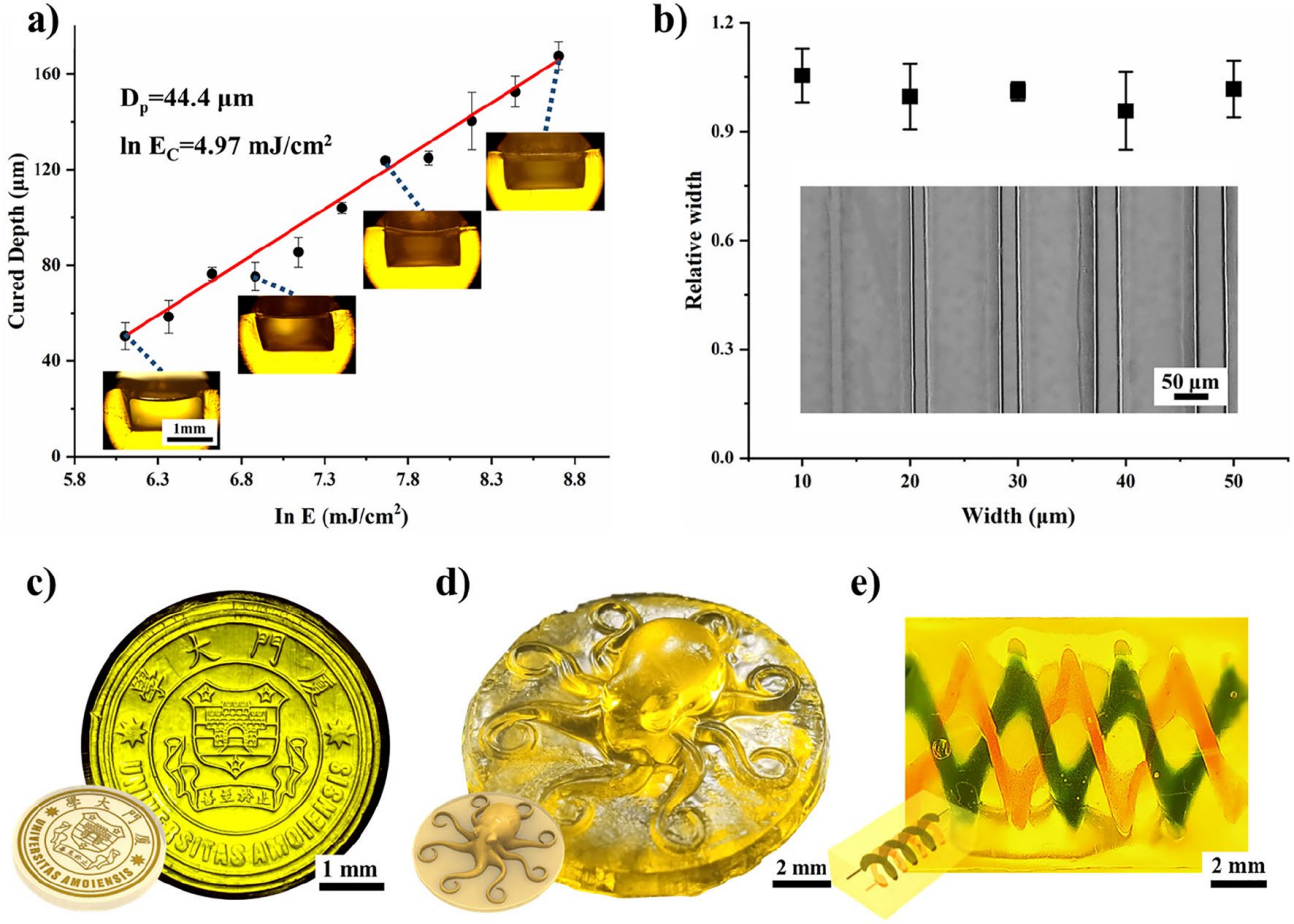
Printing parameter optimization. (**a**) Jacobs equation fitting of the curing depth of the UV-curable hydrogel solution. (**b**) Printing of a single line. (**c**) Xiamen University micro emblem and its schematic. (**d**) 3D octopus sample and its schematic. (**e**) Printed sample with double-helical flow channels.

To achieve high-resolution printing, the optimal printing parameters for the UV-curable hydrogel solution were investigated. Based on the Beer-Lambert law, the curing behavior of photocurable inks can be described by Jacobs equation^43,47^:

1 $${C_d} = {D_p}ln(E) \, - {D_p}ln({E_c})$$

where *C*_*d*_ is the depth of cure at a given exposure *E*, *D*_*p*_ is the transmission depth of UV-curable solutions, and *E*_*c*_ is the critical exposure intensity. Printing hollow structures with a transverse channel using different energy densities inputted on the top layer was performed (Supplementary Fig. S3). The thickness of the top layer film of the channel was measured to characterize the cured depth of the hydrogel solution, and the relationship between the cured depth and the energy density was fitted (Fig. 3a, *D*_*p*_ = 44.4 μm, ln(*E*_*c*_) = 4.97 mJ/cm^2^). According to this model, the ideal exposure time for 10–40 μm thick film is 4–6 s.

Utilizing these optimized process parameters, micro lines with a width ranging from 10–50 μm (Fig. 3b) were printed, achieving the upper limit resolution of the DLP printer. The application of a higher precision UV source could further improve the resolution. Furthermore, hydrogel samples with complex 2D/3D patterns were printed, including the Xiamen University emblem (Fig. 3c) and a 3D octopus (Fig. 3d), which reproduced the details of the circular design and characters with fidelity.

To further validate the printability of the material, a flexible sample containing double-helix internal channels was printed with a diameter of 1 mm (Fig. 3e). The two channels were infused with different liquids, which demonstrated smooth flow without any obstruction (Supplementary Video S1). These features endow this hydrogel formula with the capability to manufacture integrated flexible and complex 3D microfluidic chips.

### Mechanical properties of the hydrogels with adjustable modulus

Alginate in the hydrogel formula formed an adjustable ion crosslinking network in the environment of multivalent cations. Different multivalent ions exhibited varying effects on crosslinking alginate, such as Na^+^, Ca^2+^, Sr^2+^, Ba^2+^, Al^3+^, or Fe^3+^^37,48^. Besides, the concentration of the cations was also related to the crosslinking density of the polymer chains^49^. By controlling the Fe^3+^ concentration in the ion bath, the crosslinking density of the PAAm-Alg double network hydrogel was manipulated, which allowed Young's modulus adjustment to hydrogel samples. After Fe^3+^ ion bath treatment, the color of the samples intensifies with the rising Fe^3+^ concentrations.

**Figure 4:**
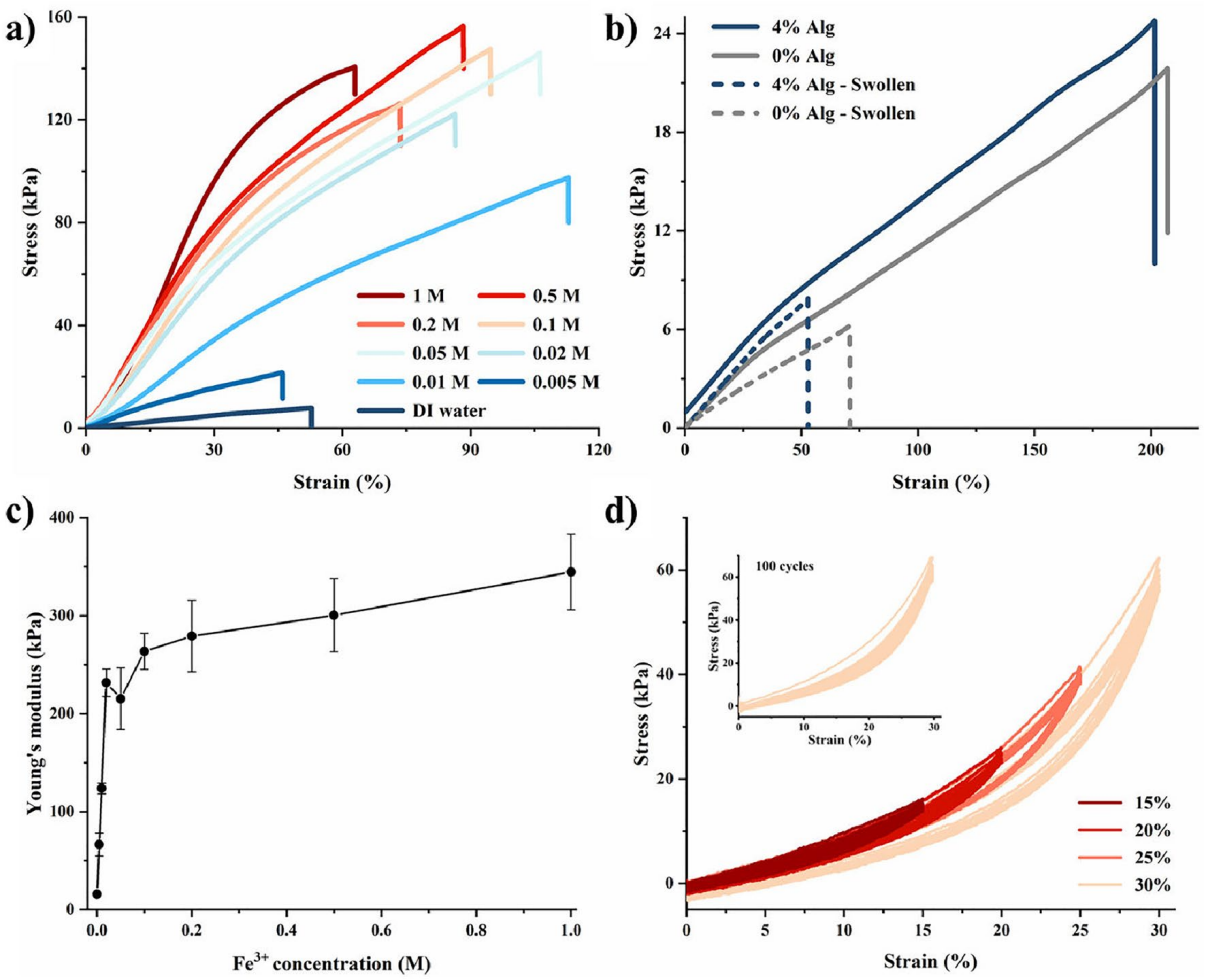
Mechanical properties of Fe^3+^-treated PAAm-Alg hydrogel samples. (**a**) Stress-strain curves of hydrogel samples treated with 0-1 M Fe^3+^ ion bath treatment. (**b**) Stress-strain curves of untreated (0, 4 wt% Alg) hydrogel samples, where the solid line represents the sample without swelling and exhibits significantly higher fracture strain than the swollen samples. (**c**) Relationship between Fe^3+^ concentration in the ion bath and the Young's modulus of the treated samples. (**d**) Cyclic tensile testing (with 15%, 20%, 25%, and 30% strain) of hydrogel samples treated with 0.1 M ion bath, with the top left inset showing the sample after 100 cycles of 30% strain.

The introduction of Alg into the hydrogel formula led to a subtle increase of modulus in untreated samples pre- and post-swelling (Fig. 4b), due to the physical double network hydrogels of PAAm-Alg^50^. Following the Fe^3+^ ion bath treatment, the hydrogel modulus exhibited a proportional increase with the ascending ion concentration, ranging from 15.8 to 345 kPa (Fig. 4a). Such a large modulus adjustable range allows the printed hydrogel scaffold to simulate the modulus of almost all human soft tissues. As shown in Fig. 4c and Supplementary Fig. S4, the elastic deformation portion of the stress–strain curves in Fig. 4a was used to calculate Young’s modulus of hydrogel samples, revealing its relationship with the Fe^3+^ concentration in the ion bath. The modulus increased rapidly and proportionally with the ion concentration, which indicated a rise in crosslinking density. However, with further increasing the Fe^3+^ ion concentration (> 0.1 M), the modulus value gradually flatten. This deceleration was attributed to a reduction in ion crosslinking sites within the alginate segments. Cyclic stretching tests on hydrogel samples (0.1 M Fe^3+^) showed that the stress–strain curves of the samples had good repeatability under 15–30% strain (Fig. 4d). However, when producing higher modulus hydrogel samples, the enhancement becomes significantly limited after exposure to higher concentration Fe^3+^ ion baths using the current hydrogel formula.

### Patterned tissue induced by hydrogel scaffolds

Various soft tissues within the human body exhibit inherent orientations, such as the organization of cardiac tissue and the intricate patterns in vascular networks. Utilizing patterned hydrogel scaffolds offers a promising method for inducing well-organized or patterned tissue growth. The cell viabilities of hydrogels crosslinked with 0 M and 1 M Fe^3+^ were 1.01 and 0.98, as shown in Supplementary Fig. S6, compared to the control group, indicating no obvious toxicity. However, the surface of PAAm-Alg hydrogel samples lacks protein segments that facilitate cell adhesion. To improve cell adhesion to the hydrogel scaffolds, it is necessary to immerse the printed scaffolds in a porcine gelatin solution.

**Figure 5:**
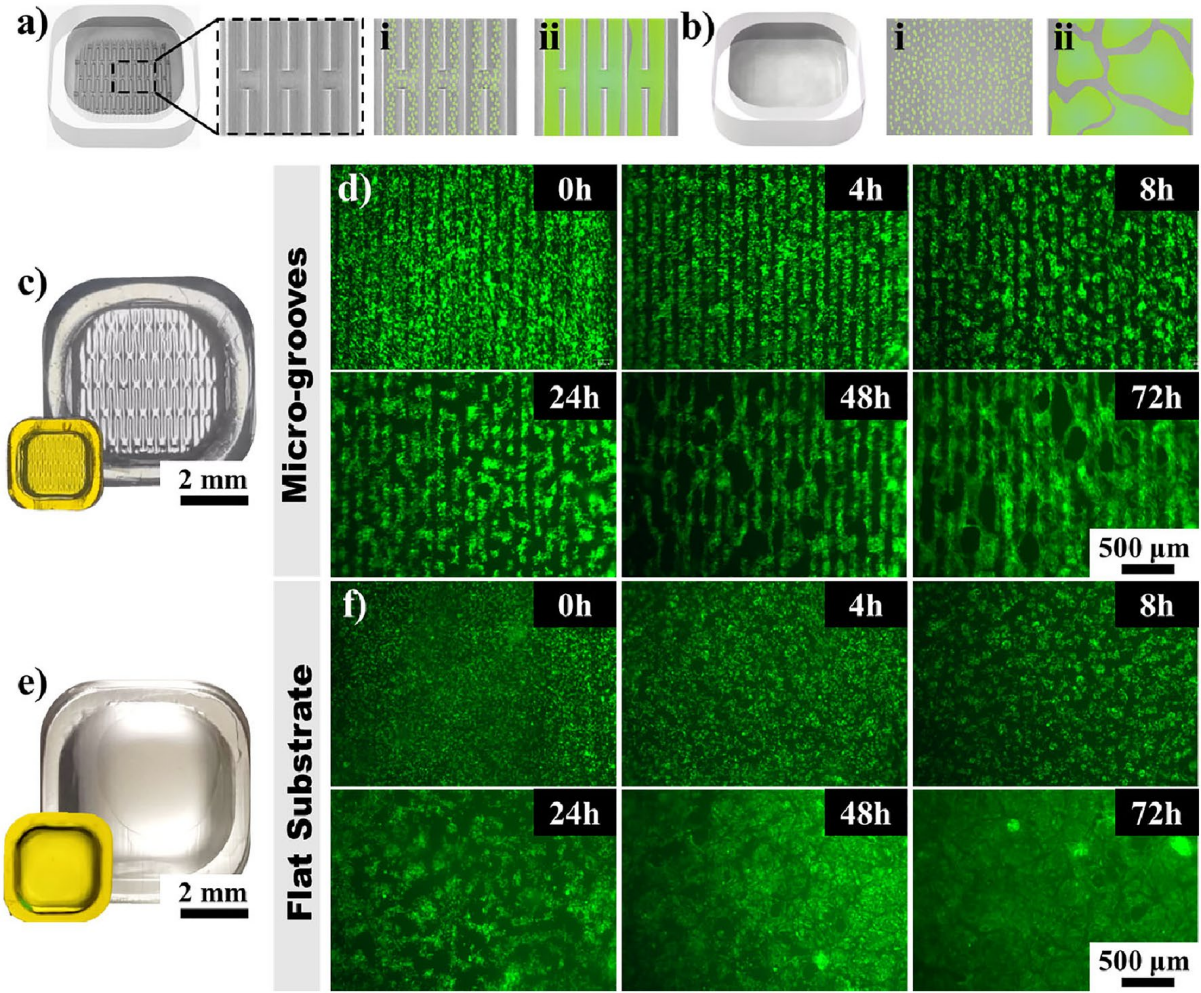
Fluorescent cardiomyocytes were cultured on cardiac tissue scaffolds. (**a**) Schematic illustration of the cultivation process on the scaffold with H-shaped grooves: (i) Cell seeding in H-shaped grooves, (ii) Induction of cardiac tissue by grooves. (**b**) Schematic illustration of cultivation process on the flat substrate: (i) Cell seeding on a flat substrate, (ii) Formation of cardiac tissue clusters. (**c**) Cardiac tissue hydrogel scaffold with H-shaped grooves, images of the printed sample and the swollen scaffold. and (**e**) flat substrates. Observing cardiac tissue cultivates on (**d**) scaffolds with H-shaped grooves and (**f**) flat substrates at 0, 4, 8, 24, 48, and 72 h reveals distinct patterns. In the H-shaped grooves, tissues were induced to form an organized sheet. On the flat substrate, cardiomyocytes gradually aggregate, forming noticeable boundaries between clusters.

Cardiac tissue scaffolds with periodic H-shaped grooves (width of 100 μm, depth of 200 μm, gap of 110 μm) were manufactured. Upon immersion in DI water, the printed H-shaped grooves experienced controlled expansion. To facilitate communication between induced cardiac tissues within each groove, additional vertical grooves were incorporated into the H-shaped grooves (Fig. 5a,c). This design allowed the seeding of cells onto the hydrogel scaffold, inducing the formation of a cohesive and organized tissue. Cardiac tissue is characterized by its softness, with a modulus of ~ 10 kPa^5–10^. Therefore, the cardiac tissue scaffold was prepared without Fe^3+^ bath treatment to maintain a minimum modulus of ~ 16 kPa. A mixed suspension of self-fluorescent cardiomyocyte and fibroblast cell (2:1) was seeded onto the flexible scaffolds. Due to the gravity, a considerable number of cells were settled down and distributed within the grooves. Cardiomyocytes gradually migrated and aggregated, forming organized and continuous tissue (Fig. 5a). As shown in Fig. 5d, cardiomyocytes transitioned from an initial spherical shape to an organized tissue structure induced by H-shaped grooves. The tissue in different lines was connected through the horizontal connections. Therefore, an organized mesh-like tissue was formed through topographical constraint. The analysis of tissue orientation from 0 to 72 h (Supplementary Fig. S7a) reveals that, during the 0–48 h culture period, cardiomyocytes gradually aligned under the induction of H-shaped grooves. Cells grew across the grooves in 72 h, forming a continuous cardiac tissue.

Comparatively, cardiac tissue cultured on a flat substrate (Fig. 5e) failed to form an organized structure and tended to grow in clusters (Fig. 5b,f). As shown in Supplementary Fig. S7b, the cardiac tissue exhibits an overall disordered state, with no distinct peaks indicating a predominant orientation. Those cells underwent arbitrary clustering, displaying uncontrollable boundaries between tissue clusters, which is more apparent in the stained images (Supplementary Fig. S8). The organized cardiac tissue could be driven to contract under an electric field generated by the electrical stimulation device. Under a pulse field with frequencies ranging from 0.5 to 1.5 Hz, the cardiac tissue exhibited synchronous contractions that followed the applied frequency (Supplementary Video S2). However, at 2 Hz, although the tissue demonstrated contractions, it failed to keep pace with the frequency of the electric field.

**Figure 6:**
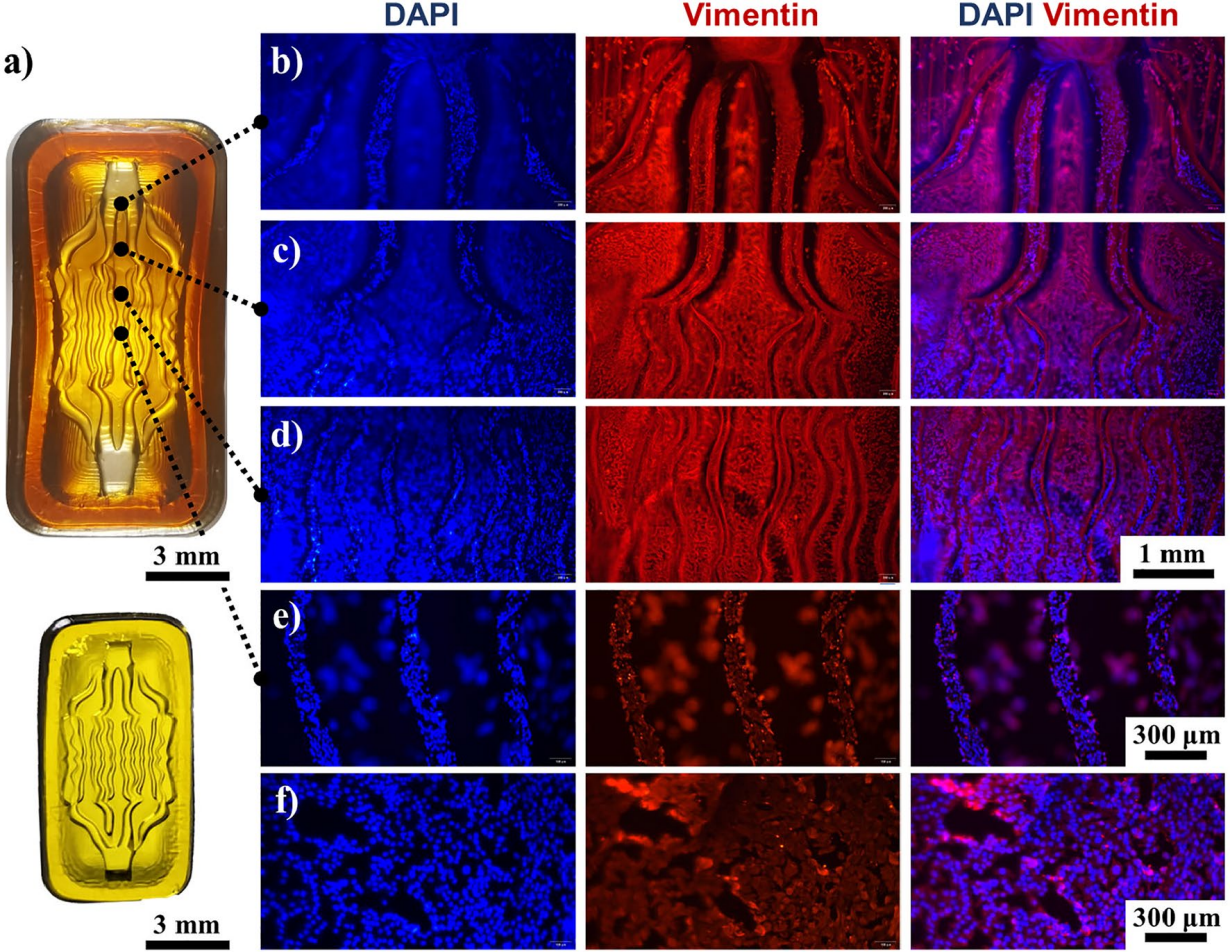
HCAECs on the vasculature-like hydrogel scaffold were stained. (**a**) The Vasculature-like hydrogel scaffold, the crosslinked sample (above) and the printed sample (below). (**b**–**e**) HCAECs seeded onto the scaffold, growing along the grooves to form vasculature-like structures. (**b**) grooves with width of 1.52 mm, (**c**) grooves with width of 0.47 mm, (**d**) grooves with width of 0.16 mm. (**e**) HCAECs were seeded at the bottom of the grooves, inducing tissue formation. (**f**) HCAECs cultured on a flat substrate exhibited disorganized distribution.

Compared with cardiac tissue, blood vessels possess a higher modulus and exhibit a more intricate macroscopic structure. Vasculature-like hydrogel scaffolds were printed and treated with 1 M Fe^3+^ to obtain Young's modulus of ~ 345 MPa, mimicking that of native blood vessels as shown in Fig. 6a. The vascular tissue scaffold was designed with semi-open channels of varying diameters. After swelling, the diameter raised from the designed 1.2, 0.3, and 0.1 mm to 1.52, 0.47, and 0.16 mm, respectively. HCAECs were seeded onto the scaffold, forming a semi-open vascular network. Tissues grown on scaffolds exhibited apparent vascular morphological features. Upon staining HCAECs across the entire scaffold, it was evident that HCAECs could be well-distributed on the scaffold and microchannels (Fig. 6b–d). Within those 0.16 mm grooves (Fig. 6e), HCAECs exhibited distinctive vascular pattern. Conversely, endothelial cells cultured on a substrate devoid of grooves did not exhibit any noticeable pattern (Fig. 6f).

## Conclusions

In conclusion, a simple and versatile two-step process, combining DLP and post-treatment. Ion bath treatment is performed after the DLP printing. Using a single AAm-Alg hydrogel formula, hydrogel structures with biocompatible, high-resolution, and adjustable modulus can be fabricated. This two-step process makes bioprinting more cost-effective and straightforward. The UV-curable hydrogel solution exhibited excellent printability, achieving a printing feature of 10 μm (limited by the resolution of the light source) that resembles the characteristic morphology of biological tissues. The ion bath treatment enables a wide range of modulus adjustments (15.83–344.67 kPa) in a simple way, with the ability to customize the modulus of hydrogel samples. Patterned hydrogel scaffolds with low or high moduli were respectively designed and manufactured for cultivating cardiac and vascular tissue. The cultivated tissues were induced by the scaffold morphology, even resembling a biomimetic multilevel vascular network. The cultured organized cardiac tissue exhibits synchronous beating under electrical stimulation. We believe that this two-step process provides an easily feasible method for manufacturing tailored scaffolds for a broad range of tissues in the human body.

### Supplementary Information

Supplementary Information.

Supplementary Video 1.

Supplementary Video 2.

## Data Availability

All data analyzed during this study are included in this published article.
