# Supplementary material for: Digital light processing printed hydrogel scaffolds with adjustable modulus
Source: Sci Rep. 2024 Jul 8;14:15695. doi: 10.1038/s41598-024-66507-x (PMC11231320; doi:10.1038/s41598-024-66507-x)
Supplement: Supplementary file 1 — Supplementary Information. [file 41598_2024_66507_MOESM1_ESM.pdf]

Supplementary Material

Digital Light Processing Printed Hydrogel Scaffolds with Adjustable Modulus

Feng Xu[1], Hang Jin[1], Huiquan Wu[1], Acan Jiang[1], Bin Qiu[1], Lingling Liu[1], Qiang Gao[2], Bin Lin[2 3], Weiwei Kong[2 3], Songyue Chen[1], and Daoheng Sun[1]**

[1] Pen-Tung Sah Institute of Micro-Nano Science and Technology, Xiamen University, Xiamen 361102, China

[2] Guangdong Provincial People's Hospital, Guangzhou, 510080, P.R. China

[3] Guangdong Beating Origin Regenerative Medicine Co. Ltd., Foshan, Guangdong 528231, China

* Author to whom any correspondence should be addressed.

E-mail: s.chen@xmu.edu.cn (S.C.); sundh@xmu.edu.cn (D.S.)

S1. Printability and Swelling Behaviors of the Hydrogel Formula

The utilization of digital light processing (DLP) technology in this study allowed for the efficient filling of voids with a hydrogel solution containing 4 wt% alginate, due to its optimal viscosity. The increased fabrication speed of the hydrogel sample enabled by the low viscosity of the solution helped mitigate damage caused by swelling during the printing process. Moreover, the viscosity of the hydrogel solution decreased with increasing temperature, and the viscosity of the 6 wt% hydrogel solution at 60°C was comparable to that of the 4 wt% hydrogel solution at room temperature. Although the 4 wt% hydrogel solution was selected as the final printing ink in this study, it should be noted that higher percentages of alginate could be manufactured at temperatures above ambient.

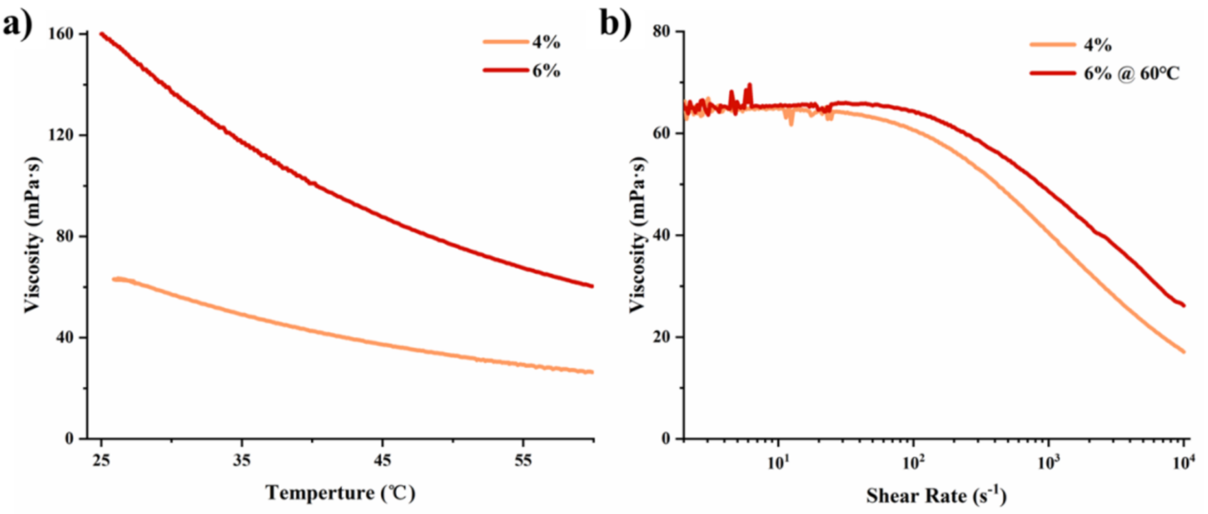

Figure S1. a) the viscosity of hydrogel solution containing 4 wt%, 6 wt% alginate over a temperature range of 25℃-60℃ at 10 s^{-1}. b) the rheological properties of hydrogel solution containing 4 wt% alginate at room temperature compared to hydrogel solution containing 6 wt% alginate at 60°C.

The swelling behavior of hydrogel samples with varying alginate concentrations (0-6 wt%) was investigated in response to different Fe^{3+} ion concentrations. As shown in Fig. S2, the deformation of hydrogel samples decreased with increasing alginate concentration. However, the effect of ion concentration on the deformation of the hydrogel samples became less pronounced as the ion concentration increased beyond 0.005M. This suggests that the alginate content is the primary factor affecting the deformation of hydrogel samples in an ion bath with Fe^{3+} concentrations above 0.005M.

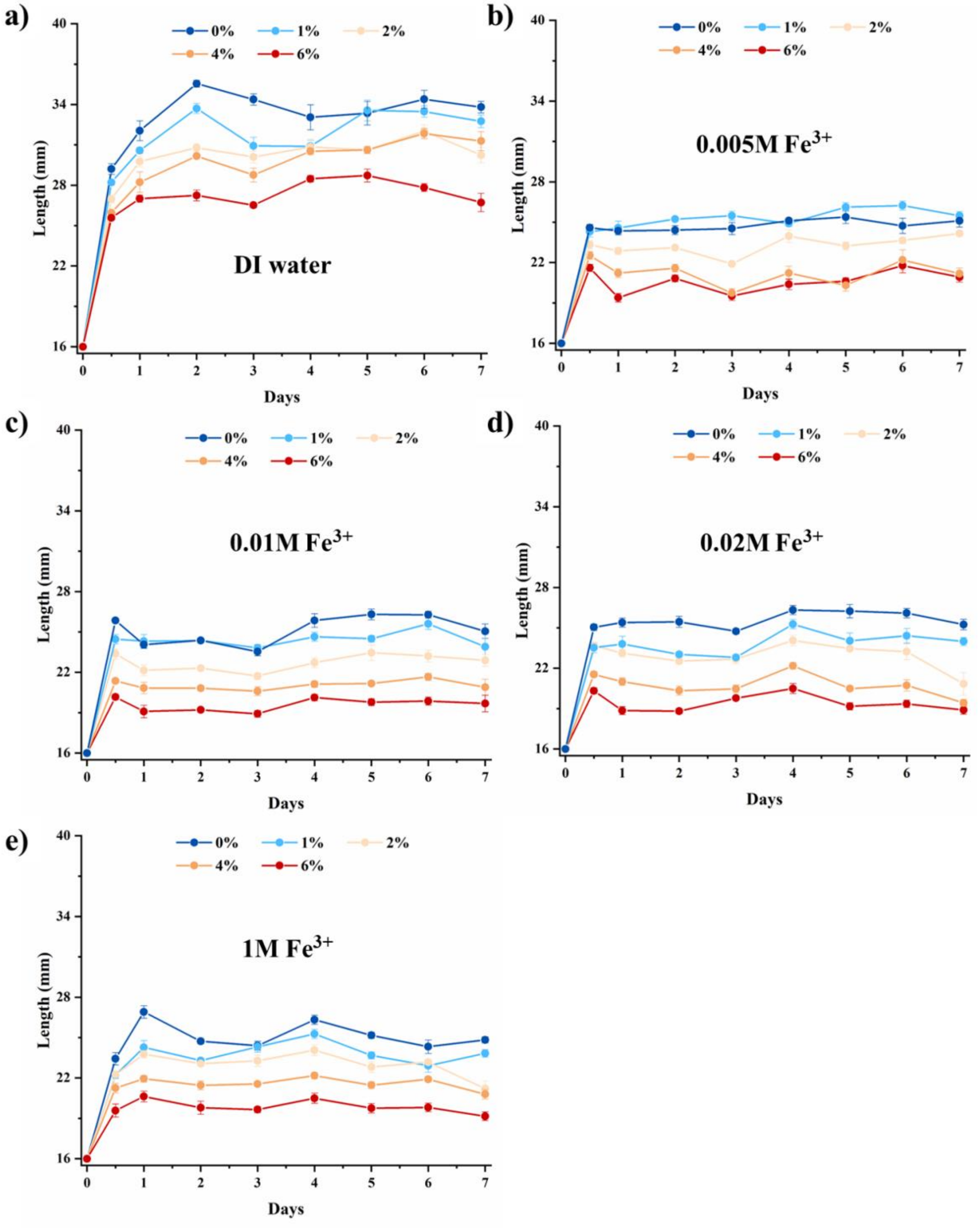

Figure S2. Swelling characteristics of the hydrogel samples with different ratios. a) sample not crosslinked, sample crosslinked in b) 0.005M Fe^{3+}, c) 0.01M Fe^{3+}, d) 0.02M Fe^{3+}, e) 1M Fe^{3+} ion baths.

S2. Optimization of DLP Parameters

To determine the relationship between the depth of cure and energy density, the energy density was adjusted by controlling the exposure time at a fixed power (P = 43.1 mW/cm²). The upper surface of the channel was measured as the depth of cure corresponding to its specific energy density. When the exposure time was less than 9 seconds, the depth of cure was too shallow, causing the elastic force of the film to be insufficient to overcome the surface tension of the solution, preventing it from escaping from the channel surface (Fig. S3a). As a result, the thickness of such films could not be measured, and the curing depths of exposure times between 10 seconds and 20 seconds were used for the fitting curve.

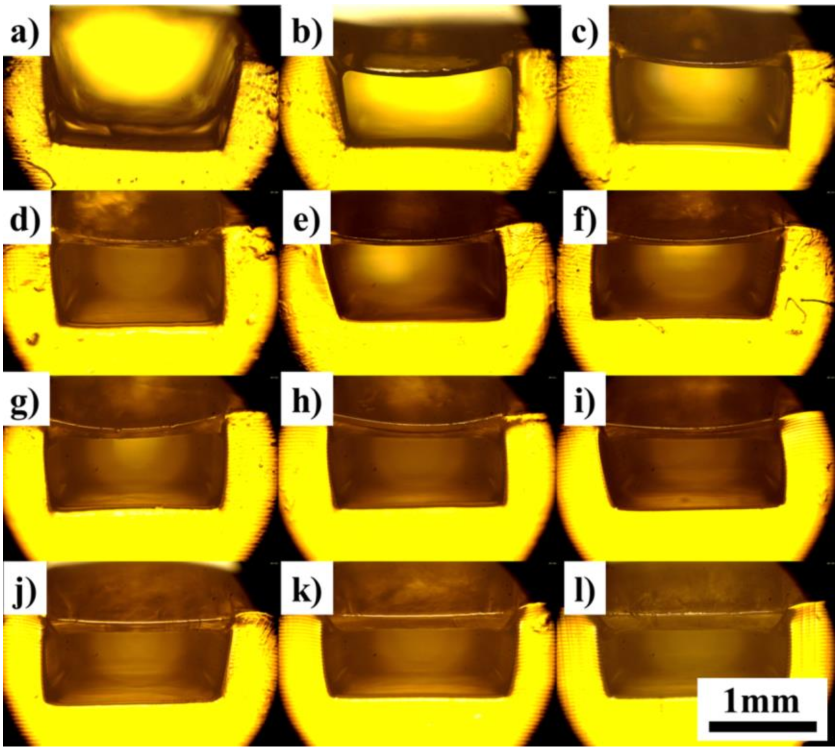

Figure S3. Depth of cure of UV curable hydrogel solution at different exposure times @P=43.1 mW/cm^2, the exposure times in Figure a-l correspond to 9-20s.

S3. Mechanical Properties of the Hydrogels with Adjustable Modulus

Hydrogel samples containing 4 wt% alginate crosslinked in Fe^{3+} ion bath concentrations of 0.005M, 0.01M, 0.02M, 0.05M, 0.1M, 0.2M, 0.5M, and 1M. The stress-strain curves of samples were tested, as shown in Fig. S4.

Hydrogel samples were washed in a 40% ethanol solution for 15 minutes, followed by crosslinking

with 0.1 M Fe^{3+}. The stress-strain curves of the hydrogel samples before and after treatment showed no significant changes (Fig. S5a), indicating that brief ethanol solution washing did not disrupt the hydrogel sample. However, gelatin coating forms a polymer layer on the surface of the hydrogel samples. The interaction between gelatin and the hydrogel results in the formation of a semi-interpenetrating polymer network (semi-IPN), which improves the modulus (increase from 13 kPa to 17 kPa) of the non-crosslinked hydrogel (Fig. S5b).

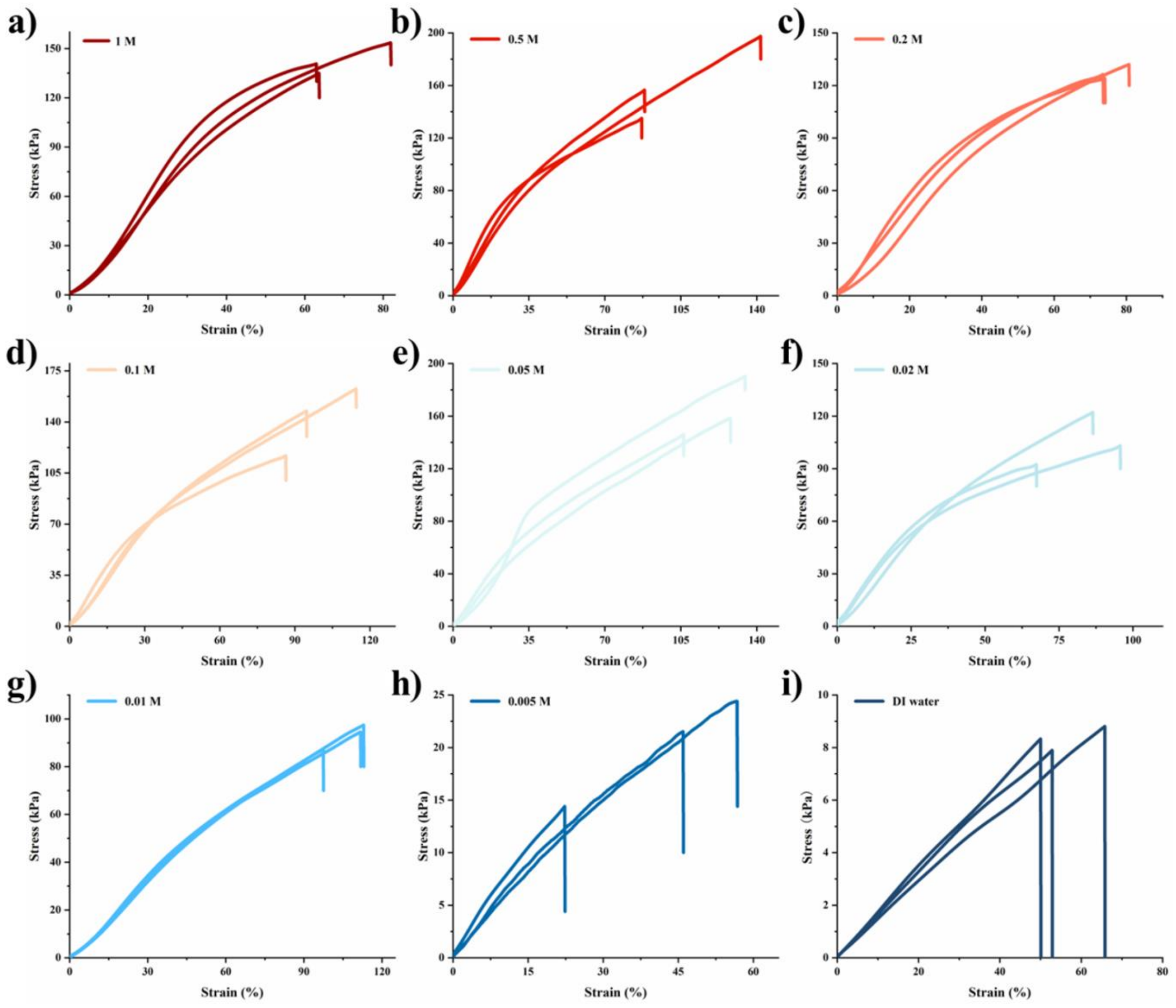

Figure S4. Stress-strain curves for tensile test specimens. The stress-strain behavior of hydrogel samples crosslinked in ion baths with varying Fe^{3+} concentrations a) 1M, b) 0.5M, c) 0.2M, d) 0.1M, e) 0.05M, f) 0.02M, g) 0.01M, h) 0.005M, and i) DI water.

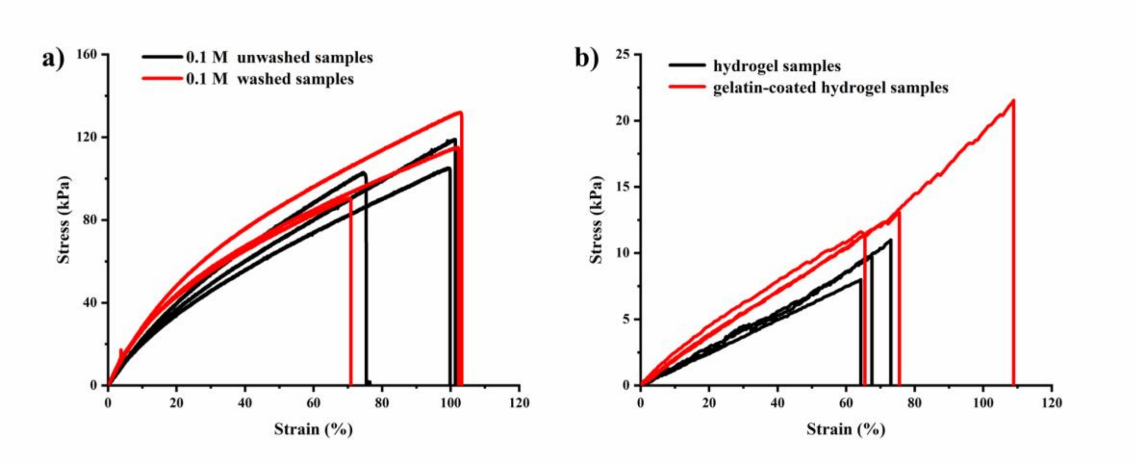

Figure S5. Impact of sample immersion in a 40 wt% ethanol solution and porcine gelatin. a) The stress-strain behavior of hydrogel samples with or without washing in 40 wt% ethanol solution followed by crosslinking in 0.1 M Fe^{3+} ion baths. b) The stress-strain behavior of hydrogel samples before and after gelatin coating.

S4. Patterned Tissue Induced by Hydrogel Scaffolds

Biocompatibility experiments were conducted using samples with varying degrees of crosslinking. Testing revealed that hydrogels crosslinked with Fe^{3+} at concentrations ranging from 0 to 1M exhibited no significant toxicity compared to the control group (Fig. S6). This indicates that the PAAm-Alg hydrogel samples themselves are non-cytotoxic and that the system, following Fe^{3+} crosslinking, remains relatively stable without causing substantial harm to the cells.

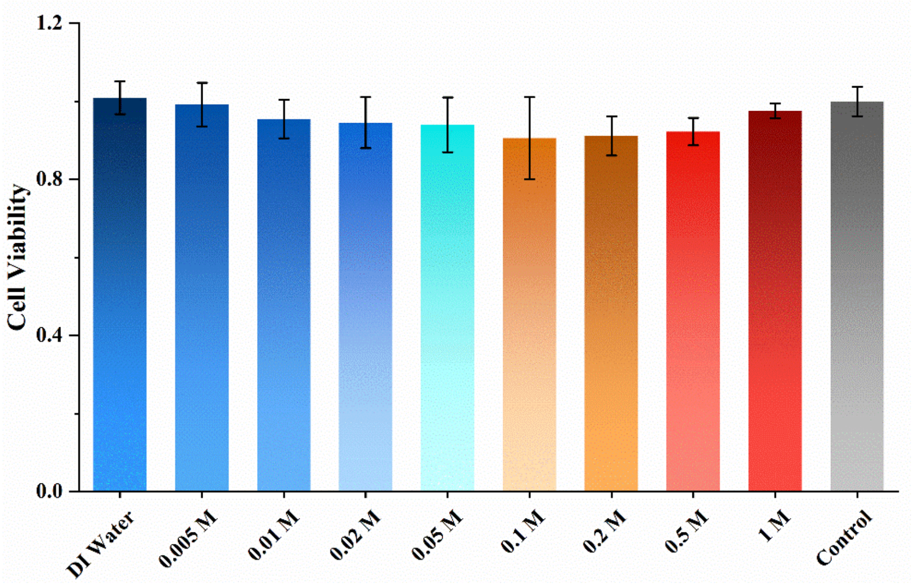

Figure S6. Biocompatibility experiments, the cell viability is tested by a CCK-8 kit and normalized by the value of the

control group.

Upon staining and observation, a distinct and organized tissue formation was evident within the microgrooves. Cardiac tissue aligned along the H-shaped grooves and the interconnectedness between parallel grooves was densely populated with cells, thus integrating the tissue into a cohesive whole (Fig. S8a-h). In contrast, cardiac tissue exhibited a fragmented structure on flat substrates, with clear boundaries between the individual clusters (Fig. S8i-p).

The OrientationJ plugin in ImageJ was used to quantify the orientation of cardiac tissue cultured on hydrogel scaffolds [1]. To compare the statistical data from different images (Fig. S7a, b), the data were normalized using the following formula [2]:

$$Intensity\ (x) = \frac{D_x}{D_{min}} - 1$$

where *Intensity(x)* is the normalized intensity at angle *x*, D_x is the measured value at angle $_x$, and D_{min} is the minimum value among the measured data. An angle of 90° corresponds to the orientation of the H-shaped grooves. Cardiac tissue cultured on micro-grooves hydrogel scaffolds showed significantly more alignment compared to those cultured on flat substrates.

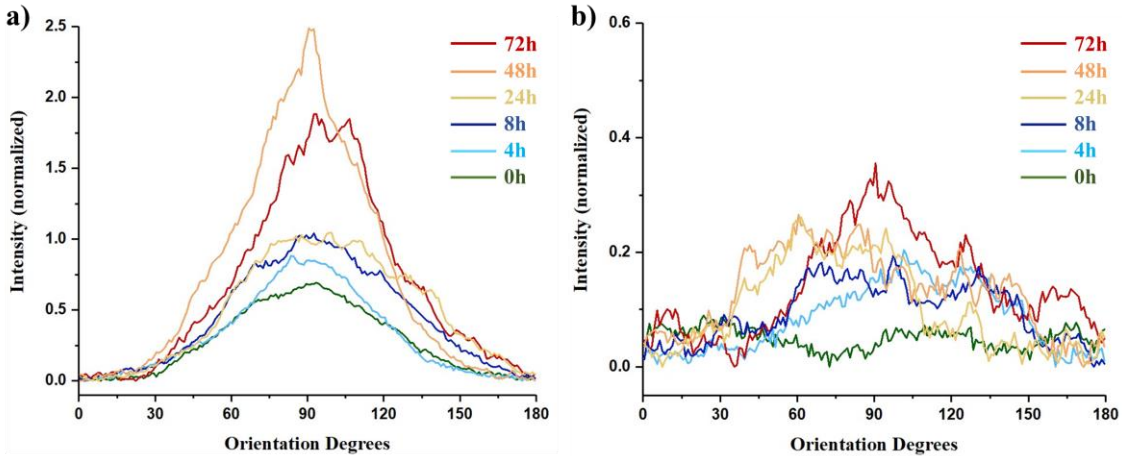

Figure S7. Cardiac tissue directional analysis. a) The orientation of cardiac tissue on the hydrogel scaffold with micro-grooves in relation to the number of days in culture. b) The orientation of cardiac tissue on flat hydrogel substance in relation to the number of days in culture.

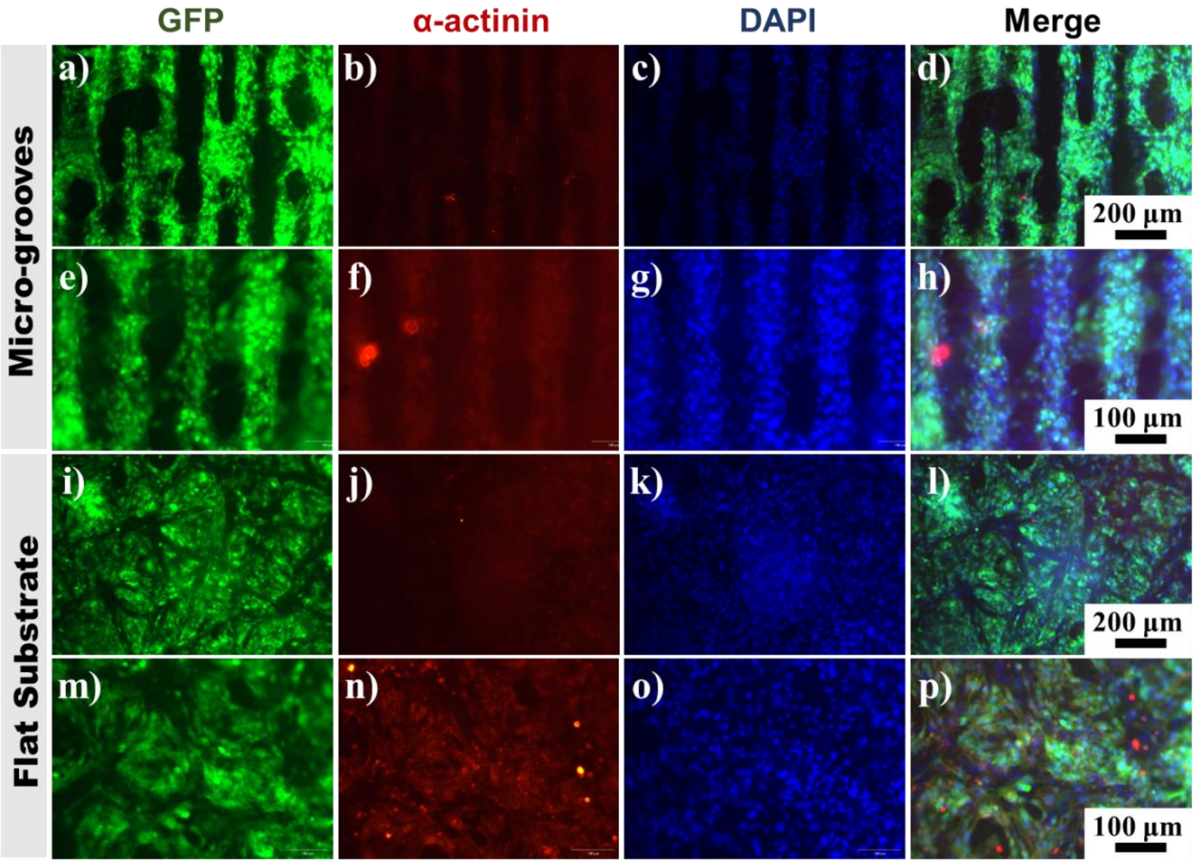

Figure S8. After 3 days of culture, cardiac tissue was immunofluorescently stained on flexible scaffolds with periodic microgrooves (a-h) and flat substrates (i-p). Fluorescence signals showed GFP (green) from fluorescent cardiomyocytes, protein α-actinin (red), and cell nuclei (blue). d) Longitudinal and transverse microgrooves induced H-shaped connections within the tissue, maintaining organization and enhancing information transfer. h) Longitudinal grooves guided orderly cardiac cell arrangement. l, p) Cardiomyocytes cultured on flat substrates exhibited noticeable aggregation and distinct boundaries.

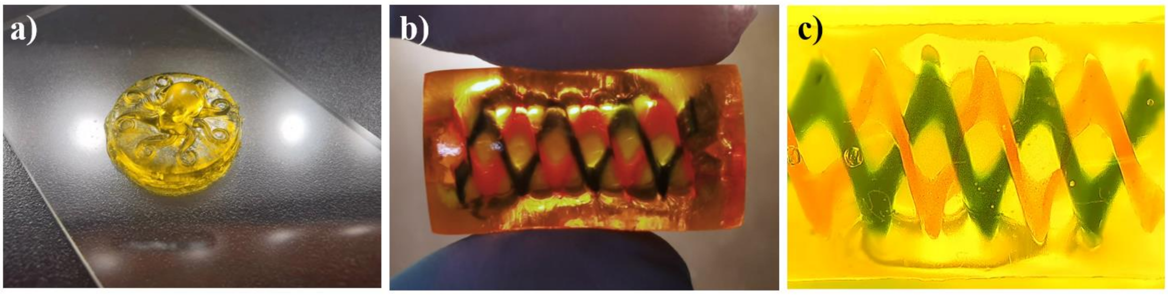

Figure S9. The original photo taken by Huawei Mate 30 mobile phone a) 3D octopus sample; b-c) Printed sample with double-helical flow channels

References

1 Wu, J.-P. *et al.* High-resolution study of the 3D collagen fibrillary matrix of Achilles tendons without tissue labelling and dehydrating. JMic 266, 273-287, doi:10.1111/jmi.12537 (2017).

2 Ayres, C. E. et al. Measuring fiber alignment in electrospun scaffolds: a user's guide to the 2D fast Fourier transform approach. Journal of Biomaterials Science-Polymer Edition 19, 603-621, doi:10.1163/156856208784089643 (2008).
